# State-Dependent Entrainment of Prefrontal Cortex Local Field Potential Activity Following Patterned Stimulation of the Cerebellar Vermis

**DOI:** 10.3389/fnsys.2019.00060

**Published:** 2019-10-29

**Authors:** Stéfanie A. Tremblay, C. Andrew Chapman, Richard Courtemanche

**Affiliations:** ^1^Department of Health, Kinesiology, and Applied Physiology, Center for Studies in Behavioral Neurobiology, Concordia University, Montreal, QC, Canada; ^2^Department of Psychology, Center for Studies in Behavioral Neurobiology, Concordia University, Montreal, QC, Canada

**Keywords:** oscillations, synchrony, cerebellum, vermis, prefrontal cortex, stimulation

## Abstract

The cerebellum is involved in sensorimotor, cognitive, and emotional functions through cerebello-cerebral connectivity. Cerebellar neurostimulation thus likely affects cortical circuits, as has been shown in studies using cerebellar stimulation to treat neurological disorders through modulation of frontal EEG oscillations. Here we studied the effects of different frequencies of cerebellar stimulation on oscillations and coherence in the cerebellum and prefrontal cortex in the urethane-anesthetized rat. Local field potentials were recorded in the right lateral cerebellum (Crus I/II) and bilaterally in the prefrontal cortex (frontal association area, FrA) in adult male Sprague-Dawley rats. Stimulation was delivered to the cerebellar vermis (lobule VII) using single pulses (0.2 Hz for 60 s), or repeated pulses at 1 Hz (30 s), 5 Hz (10 s), 25 Hz (2 s), and 50 Hz (1 s). Effects of stimulation were influenced by the initial state of EEG activity which varies over time during urethane-anesthesia; 1 Hz stimulation was more effective when delivered during the slow-wave state (Stage 1), while stimulation with single-pulse, 25, and 50 Hz showed stronger effects during the activated state (Stage 2). Single-pulses resulted in increases in oscillatory power in the delta and theta bands for the cerebellum, and in frequencies up to 80 Hz in cortical sites. 1 Hz stimulation induced a decrease in 0–30 Hz activity and increased activity in the 30–200 Hz range, in the right FrA. 5 Hz stimulation reduced power in high frequencies in Stage 1 and induced mixed effects during Stage 2.25 Hz stimulation increased cortical power at low frequencies during Stage 2, and increased power in higher frequency bands during Stage 1. Stimulation at 50 Hz increased delta-band power in all recording sites, with the strongest and most rapid effects in the cerebellum. 25 and 50 Hz stimulation also induced state-dependent effects on cerebello-cortical and cortico-cortical coherence at high frequencies. Cerebellar stimulation can therefore entrain field potential activity in the FrA and drive synchronization of cerebello-cortical and cortico-cortical networks in a frequency-dependent manner. These effects highlight the role of the cerebellar vermis in modulating large-scale synchronization of neural networks in non-motor frontal cortex.

## Introduction

There has been growing evidence supporting cerebellar involvement in cognitive and affective functions (Hoppenbrouwers et al., 2008; Strick et al., 2009; Bostan et al., 2013). The cerebellum may promote synchronization of large-scale networks and influence extra-cerebellar networks through multiple cortical and subcortical projections (Courtemanche et al., 2013; Farzan et al., 2016). The cerebellum shows regional variations in its relatively uniform circuitry (Cerminara et al., 2015) and, through its wide connectivity, can modulate specific circuits involved in motor control, cognition, and affect (Schmahmann, 2004, 2019). Because of its contribution to several neurological disorders and its extensive connectivity with extra-cerebellar structures, the cerebellum has been used as a therapeutic target for non-invasive stimulation techniques such as transcranial magnetic stimulation (TMS) and transcranial direct current stimulation (tDCS) (Hoppenbrouwers et al., 2008; van Dun et al., 2017, 2018). Stimulation of the vermis, the most medial region of the cerebellum, results in positive effects on cognition and mood associated with modulation of frontal oscillations (Schutter et al., 2003; Schutter and van Honk, 2006, 2009; Demirtas-Tatlidede et al., 2010). Stimulation of the fastigial nucleus (FN) following middle cerebral artery occlusion and chronic mild stress in rats also improves neuroprotection by suppressing death of cerebellar Purkinje cells and alleviates depressive-like behaviors (Zhang et al., 2017). Stimulation of the FN also affects local field potential (LFP) oscillations in the frontal cortex of anesthetized cats, where high frequency stimulation attenuates slow rhythms, and enhances 20–40 Hz oscillations (Steriade, 1995). Low frequency FN stimulation (1 Hz) was also shown to inhibit epileptogenic activity in the rat (Wang et al., 2008), while stimulating lateral cerebellar projections at 2 Hz has been shown to rescue medial frontal cortex delta activity in a rat model of schizophrenia (Parker et al., 2017).

Brain imaging studies in humans have demonstrated functional connectivity between the cerebellum and prefrontal cortex (PFC) (O’Reilly et al., 2009; Buckner et al., 2011; Sang et al., 2012; Farzan et al., 2016). The underlying connections have also been well characterized in non-human primates (Kelly and Strick, 2003; Strick et al., 2009) and rodents (Watson et al., 2009, 2014; Suzuki et al., 2012) using neuroanatomical and electrophysiological approaches. In the urethane-anesthetized rat, stimulation of the medial PFC (prelimbic cortex; PrL) evoked responses in the contralateral vermis (lobule VII), while stimulation of the fastigial nucleus resulted in evoked potentials in the PrL, indicating reciprocal long-range interactions between the medial PFC and medial cerebellum (Watson et al., 2009, 2014). Watson et al. (2014) also observed synchronous LFP activity in the theta range (5–10 Hz) between the fastigial nucleus and PrL during active locomotion and at rest.

Coherent synchronization of rhythmic neuronal population activity between distant cortical regions is thought to reflect mechanisms that enhance communication between structures, and that coordinate contributions of brain regions to sensorimotor integration and cognitive function (Engel et al., 2001; Fries, 2015). There is growing interest in understanding how cerebello-cortical network interactions synchronize to modulate higher-order functions. The dorsolateral PFC and the vermis have been implicated in the pathogenesis of several neurological disorders (Baxter et al., 1989; Andreasen et al., 1996; Maeda et al., 2000a; Andreasen and Pierson, 2008; Koenigs and Grafman, 2009; Fatemi et al., 2012). Anatomical evidence in monkeys (Kelly and Strick, 2003) and functional connectivity studies in humans (Buckner et al., 2011; Farzan et al., 2016) have demonstrated pathways mediating communication between the vermal lobule VII and dorsolateral PFC. However, little is known concerning the frequencies of activity that promote coherent LFP oscillations in these structures most effectively, and to what extent the induction of coherent LFP activity may depend on the initial oscillatory state.

In the current study, we investigated the effects of various frequencies of cerebellar vermis stimulation on the power and coherence of LFP oscillations in Crus I/II of the right lateral cerebellum (RCb) and bilateral dorsolateral PFC (frontal association area; FrA) in the urethane-anesthetized rat. Urethane is permissive to oscillations, and results in cyclic alternations between states similar to slow-wave nREM sleep and active REM sleep (Clement et al., 2008; Ros et al., 2009). Based on previous anatomical and functional connectivity studies (Akgören et al., 1996; Kelly and Strick, 2003; Buckner et al., 2011; Farzan et al., 2016), stimulation of the vermis was expected to strongly modulate LFP activity in the FrA and in the RCb. Stimulation was delivered to the most superficial layer of the cerebellar cortex to activate Purkinje cells (PCs) that project to the fastigial nucleus, which can modulate cortical areas via the thalamus and the cerebellar hemispheres via parallel fibers (Akgören et al., 1996; Lisberger and Thach, 2013). Inhibitory interneurons would also likely be activated by stimulation, which could result in complex frequency-specific interactions (Dugué et al., 2009). The goals of this study were to (1) characterize spontaneous LFP activity in the lateral Cb and bilateral dorsolateral PFC [FrA in the rat; (Uylings et al., 2003)] as well as coherence within this network during urethane-anesthesia, (2), assess the effectiveness of different frequencies of vermal stimulation in inducing changes in power and coherence in LFP activity in the lateral Cb and FrA, and (3), determine how slow-wave and activated stages of urethane anesthesia may modulate the responsivity of the network to stimulation. Lower frequencies of stimulation were expected to have a greater impact on slow oscillatory activity and coherence, and higher frequencies were expected to drive cortical beta and gamma oscillations (Steriade, 1995; Schutter et al., 2003; Schutter and van Honk, 2006; Wang et al., 2008; Parker et al., 2017).

## Materials and Methods

### Surgery

Six adult male Sprague-Dawley rats were used in this study. The anesthesia procedures were the same as used by Frederick et al. (2014). Briefly, rats were anesthetized with a 5% isoflurane and 95% oxygen mixture, and a catheter was placed in the jugular vein. Urethane (0.8 g/ml) was then administered intravenously to maintain anesthesia, and level of anesthesia was verified by ensuring that the foot-withdrawal reflex was absent throughout the experiment. Rats were placed in a stereotaxic apparatus and a regulated heating pad and insulating blanket were used to maintain body temperature near 37°C. All procedures were in accordance with the guidelines of the Canadian Council on Animal Care and approved by the Concordia University Animal Research Ethics Committee.

**FIGURE 1:**
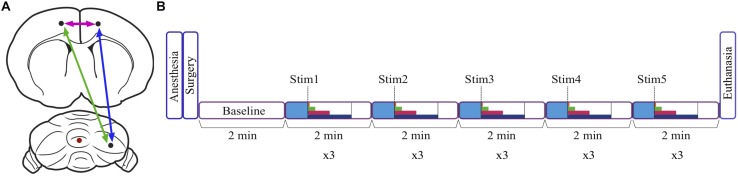
Schematic diagram of the location of stimulation and recording sites, and the experimental timeline. **(A)** Recording sites in the right cerebellum and the left and right frontal association area are represented by black dots, and the stimulation site in the cerebellar vermis is indicated by a red dot. Arrows represent coherence between sites. **(B)** After anesthetizing the animal, electrodes were inserted stereotaxically during surgery, and a baseline period of 2 min was recorded. There was also a baseline period of 30 s (blue) at the start of each 2 min trial. The duration of the stimulation depended on stimulation frequency and varied from 1 to 60 s (dark blue = 60 s, pink = 30 s, green = 10 s, orange = 2 s, dashed line = 1 s). Three trials were conducted for each of the five stimulation frequencies, and the animal was euthanized following recordings.

The skin was cut to expose the skull and a 2–2.5 mm craniotomy was performed in the occipital bone over the right cerebellar Crus I/II lobule. Holes were also drilled bilaterally over the FrA, and over the cerebellar vermis lobule VII. Bipolar electrodes, constructed from Teflon-coated stainless-steel twisted wire (125 μm tip diameter, with tips 1 mm apart in depth), were anchored to the stereotaxic apparatus. The stimulation electrode was inserted into the vermis lobule VII (AP -13.0; ML 0; V 3.3), and recording electrodes were inserted into the FrA (AP 4.7; ML ± 1.8; V 2.2). The recording electrode in the right Crus I/II was inserted at a 45° angle, 3.2 mm lateral from the midline, to a depth of 1 mm from the surface of the cerebellum (Figure 1A). The stereotaxic apparatus was grounded, and a bare stainless-steel reference electrode (5 mm long) was placed between the skull and the surface of the temporal lobe. Two monopolar recordings and one bipolar differential recording were obtained from each site. Both types of recordings can be used to record LFP activity yet electrode placement must be aligned to a dipole to obtain an optimal bipolar signal (Buzsaki et al., 2012). Bipolar recordings were thus used when the signal amplitude was optimal, otherwise monopolar recordings were used.

### Recording Procedures

Recordings in each animal were initiated with a 2 min recording of spontaneous baseline LFP activity in the RCb and FrA. LFP signals were band-pass filtered between 0.01 and 500 Hz, amplified (x1000; A-M Systems Model 1700), and digitized onto the computer’s hard drive at a sampling rate of 1024 Hz using SciWorks software (Datawave Technologies, Loveland, CO, United States). LFPs were recorded bilaterally in the FrA in three animals, in the left FrA (LFrA) in one animal, and in the right FrA (RFrA) in two animals. Each recording trial, in which a different stimulation frequency was tested, lasted 2 min. Following a 30 s baseline period, stimulation was delivered for 1–60 s, depending on the frequency of stimulation, and this was followed by a post-stimulus recording (Figure 1B). Biphasic square-wave pulses (0.1 ms duration) were delivered to the vermis using a stimulus generator (A-M Systems, Model 2100; Sequim, WA, United States). In addition to a single-pulse condition, in which pulses were delivered every 5 s for 60 s, stimulation frequencies were selected within all major frequency bands. Repeated pulses were delivered at 1 Hz (30 s duration, delta), 5 Hz (10 s, theta), 25 Hz (2 s, beta), and 50 Hz (1 s, gamma). Stimulation was delivered in ascending order (from lowest to highest frequency) in three animals and was delivered in randomized order in the other three animals. There were no significant effects of testing order on measures of power or coherence. Each frequency of stimulation was delivered at an intensity of 500, 750, and 1000 μA resulting in three trials at each stimulation frequency (two trials at each intensity were obtained in one animal; Table 1). Following recordings, animals were euthanized with an intravenous overdose of urethane.

**TABLE 1:** Recording sites, number of trials per stimulation pattern and order of stimulation pattern delivery for each animal.

**Rat**	**Recording sites**	**# trials/stim type**	**Order of stim delivery**
1	RCb, RFrA	3	Low to high frequency
2	RCb, RFrA	3	Low to high frequency
3	RCb, LFrA	3	Low to high frequency
4	RCb, LFrA, RFrA	3	Randomized
5	RCb, LFrA, RFrA	6	Randomized
6	RCb, LFrA, RFrA	3	Randomized

### Signal Processing and Analysis

Recordings were imported into MATLAB (Mathworks, Natick, MA, United States) for analysis. Signals were filtered using the function *filtfilt*, with a FIR equiripple low-pass at 250 Hz. Power spectral density analyses (short-time Fourier transform) were conducted using the spectrogram function with windows of 512 samples (0.5 s) and a 50% overlap. This resulted in good temporal resolution (0.25 s), allowing slow components of the signal to be quantified. Spectrograms were constructed to represent the frequency content of the signal as a function of time.

For coherence analysis, the filtered signals were divided into epochs of 2 s. The magnitude-squared coherence, which indicates how closely related two signals (x and y) are in power across frequencies and the consistency of the phase relationship between the two signals at each frequency, was computed for each electrode pair using the mscohere function:

$$C_{x\,y}\,\left(f\right)=\frac{|P_{x\,y}\left(f\right)|{}^{2}}{P_{x\,x}\,\left(f\right)\,P_{y\,y}\,\left(f\right)}$$

where Pxx(f) and Pyy(f) are the power spectral densities of x and y, and Pxy(f) is the cross power spectral density.

Each bipolar recording electrode provided two monopolar channels and one differential recording channel for each recording site (RCb, RfrA, and LFrA). One channel was chosen for analysis for each recording site. The differential bipolar recordings were chosen when possible, but the largest amplitude monopolar recording channel was used when similarity of the monopolar channels resulted in very low power in bipolar recordings. Coherence was calculated between RCb-LfrA (contra Cb-FrA), RCb-RfrA (ipsi Cb-FrA), and LfrA-RfrA (FrA-FrA), using the selected channels.

Power and coherence values were integrated within each frequency band: delta (Δ, 0.01–3 Hz), theta (θ, 3–8 Hz), alpha (α, 8–15 Hz), beta (β, 15–30 Hz), low gamma (low γ, 30–55 Hz), high gamma (65–80 Hz), and fast (80–200 Hz), and normalized by dividing by the number of frequency bins within each band. Frequencies between 55 and 65 Hz were left out to eliminate 60 Hz noise. Spectrograms and coherograms were inspected to assess the time-course of changes following stimulation, and power and coherence values were averaged across periods of 6 s. This resulted in five pre-stimulation periods (30 s period), 4 post-stimulation periods for single-pulse stimulation, and 8 post-stimulation periods for all other conditions. The relative changes in post-stimulation values of power and coherence were calculated from the mean pre-stimulation values for each period.

**FIGURE 2:**
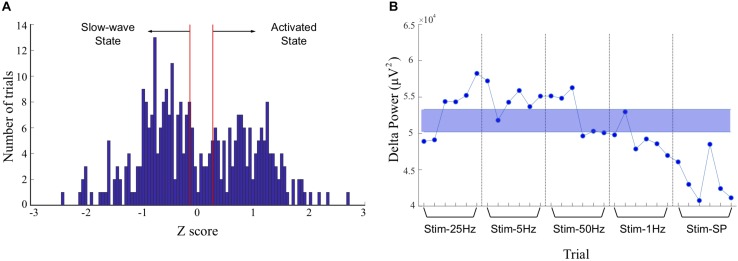
Discrimination of trials recorded during slow-wave and activated states. **(A)** Histogram of z-scores of power in the delta band in cortical recordings during the 30 s baseline period, for all trials. The peak on the right represents greater delta power during the slow-wave state, and the peak on the left represents lower delta power during the activated state. The area between the red lines was identified as a transition period and corresponds to the median and to the 60th percentile of the distribution. The trials between the 50th and 60th percentiles were excluded from the stage-based analyses. **(B)** Example of trials attributed to the slow-wave or activated states in one animal (rat 5). Power in the delta band is plotted for a monopolar recording in the left frontal association area, and stimulation frequencies tested during each consecutive trial are indicated on the x-axis. Trials with values within the shaded blue area were excluded from analysis, and trials above and below the shaded areas were classified as representing the slow-wave, or activated states, respectively. SP, single-pulse.

Neocortical activity at ∼1°Hz (delta) is associated with the slow-wave state under urethane anesthesia, and low-amplitude faster cortical oscillations are present during the activated state (Clement et al., 2008). To address the impact of the state-changes during anesthesia, we divided the trials into either the slow-wave state (Stage 1) or the activated state (Stage 2) based on the amount of power in the delta band in cortical channels during the pre-stimulation period. The z-scores of cortical power in the delta band for all trials were plotted on a histogram (Figure 2A) and trials above the 60th percentile were classified as slow-wave, and trials below the 50th percentile were classified as activated state. Trials between the 50th and the 60th percentiles were considered as being in transition and were excluded from the stage analysis (see example in Figure 2B). The two subgroups of trials formed for each animal were used to determine if the stage of anesthesia has an impact on frequency-dependent effects of stimulation. Effects of stimulation did not typically outlast the 2 min trial duration, and this state-dependent analysis ensured that only trials representative of the slow-wave or activated state were included in the respective analyses.

### Statistical Analysis

Repeated-measures ANOVAs were conducted using Tibco Statistica (Dell Software, Round Rock, TX, United States). Stimulation intensity had no significant effect on power [one rat in which 2 trials per intensity per stimulation frequency were obtained: *F*_(2, 288)_ = 1.92, *p* = 0.15]. Therefore, trials of different intensities were grouped together for each stimulation frequency. The order in which the stimulation patterns were delivered also had no significant effect on measures of power [StimOrder: *F*_(1, 230)_ = 0.31, *p* = 0.860; Stim by StimOrder interaction: *F*_(4, 230)_ = 0.485, *p* = 0.747] or coherence [StimOrder: *F*_(1, 211)_ = 0.008, *p* = 0.927; Stim by StimOrder interaction: *F*_(4, 211)_ = 0.410, *p* = 0.802]. The data were therefore combined together.

For initial analyses, the dependent variables were LFP power and LFP-LFP coherence, while the independent variable was the type of stimulation. Repeated measures ANOVAs, with 7 levels of frequency bands and levels of time (1 Baseline and 4–8 Post windows, depending on the condition) as repeated measures, and stimulation type (Stim-SP, Stim-1 Hz, Stim-5 Hz, Stim-25 Hz, and Stim-50 Hz) and site (RCb, LfrA, RfrA) as categorical factors, were performed on the relative changes from baseline for LFP power. A similar analysis was done for coherence but included the pair of recording sites (contra Cb-FrA, ipsi Cb-FrA, and FrA-FrA) as a categorical factor. Results were considered statistically significant if *p* < 0.05.

To assess state-dependent differences in response to stimulation, separate repeated-measures ANOVAs for each stimulation frequency assessed changes in power or coherence in a given frequency band relative to baseline as a function of the time window and stage. These analyses were performed within each recording site for LFP power, and between each pair of recording sites for coherence (site or comparison type as categorical factor). Fisher’s *post hoc* analyses (*p* < 0.05) were used to identify which components differed in statistically significant interactions.

## Results

### Spontaneous Power and Coherence

**FIGURE 3:**
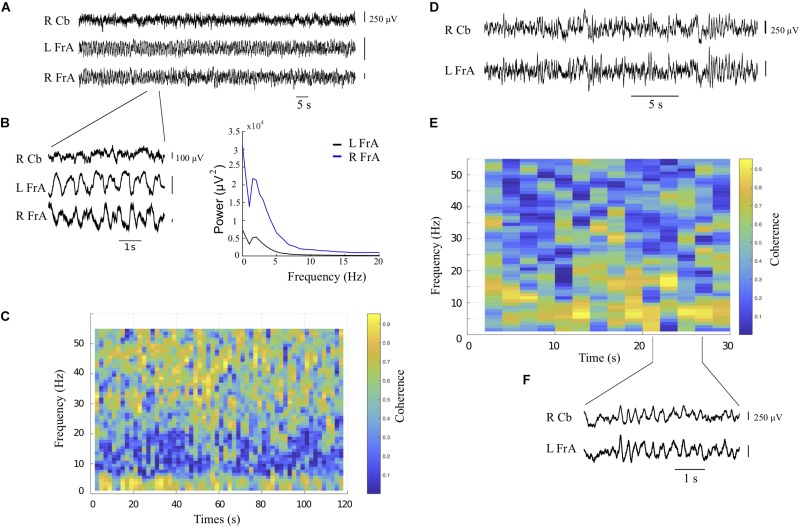
Spontaneous oscillatory activity and coherence during baseline recordings from the right cerebellum (RCb) and left and right frontal association area (LFrA, RFrA). **(A)** Local field potential (LFP) recordings are shown for each recording site (rat 4). **(B)** Expanded view of LFP signals in **(A)** shows fast activity on top of slow-wave oscillatory activity in cortical sites. The corresponding power spectra reflect dominant rhythms at 2 Hz in the LFrA and RFrA during slow-wave activity. **(C)** The coherogram for the LFrA and RFrA shows periods of coherent activity at frequencies near 2 Hz and in the low gamma band (30–50 Hz). **(D)** Additional example of LFP traces in the RCb and LFrA (rat 3, 2 recording sites). **(E)** The coherogram shows coherence at 10 Hz, in the theta/alpha bands, between the RCb and LFrA. **(F)** Expanded LFP traces from the RCb and LFrA show prominent 10 Hz activity in both sites (lines indicate the time in **E** at which recordings were obtained).

Prior to assessing the effects of cerebellar rhythmic stimulation, spontaneous LFP power within each recording site, and coherence between each pair of recording sites were evaluated. Power was typically highest in the delta band in all animals, consistent with the slow-wave activity reported in urethane-anesthetized rats (Clement et al., 2008; Frederick et al., 2014). Baseline power spectra in all sites showed peaks in the delta band at about 2 Hz. This activity co-occurred with activity in the beta, low gamma and high gamma bands, which was superimposed on the slow waves. This higher-frequency activity was not always clear in power measures but was readily evident in coherence measures. The example in Figures 3A–C shows coherent activity in both delta and low gamma bands between the left and right FrA.

LFP recordings also showed periods in which delta band activity was weaker, and there was a more broadband distribution that sometimes included periods of increased power and coherence around 8–10 Hz. The example in Figures 3D–F shows marked coherence in the alpha band (10 Hz) between the cerebellum and contralateral cortex, consistent with previous electrophysiological evidence in the cerebellum and neocortex (O’Connor et al., 2002). These periods when slow-wave activity subsides to give way to faster activity in neocortical sites are consistent with the activated state described by Clement et al. (2008).

### Effects of Stimulation

An initial analysis was used to evaluate how LFP power and coherence were modulated by different frequencies of stimulation across all recorded trials. The time by stimulation frequency interaction was significant [*F*_16_, _980__)_ = 2.28, *p* = 0.003], with 50 Hz stimulation inducing more rapid effects on power, and single-pulse stimulation inducing more delayed effects. There was also a trend for a stimulation type by site interaction [*F*_(8, 245)_ = 1.84, *p* = 0.071], with the cerebellar site differing from cortical sites in the responses to stimulation frequencies, and a significant time by site interaction [*F*_8_, _980__)_ = 2.44, *p* = 0.013] due to earlier responses of the cerebellar recording to stimulation compared to the two cortical sites. No main effects or interactions were seen for coherence in this overall analysis.

### Stage-Dependent Effects of Stimulation

The initial analysis indicated that vermal stimulation had frequency-dependent effects on LFP activity in both cerebellum and cortical sites, but baseline LFP activity differed markedly between the slow-wave state and the activated states. We therefore separated trials between the slow-wave (Stage 1) and activated (Stage 2) states and conducted ANOVAs to evaluate specific effects of stimulation frequency during each stage, on LFP and coherence measures in specific sites for each frequency band.

**FIGURE 4:**
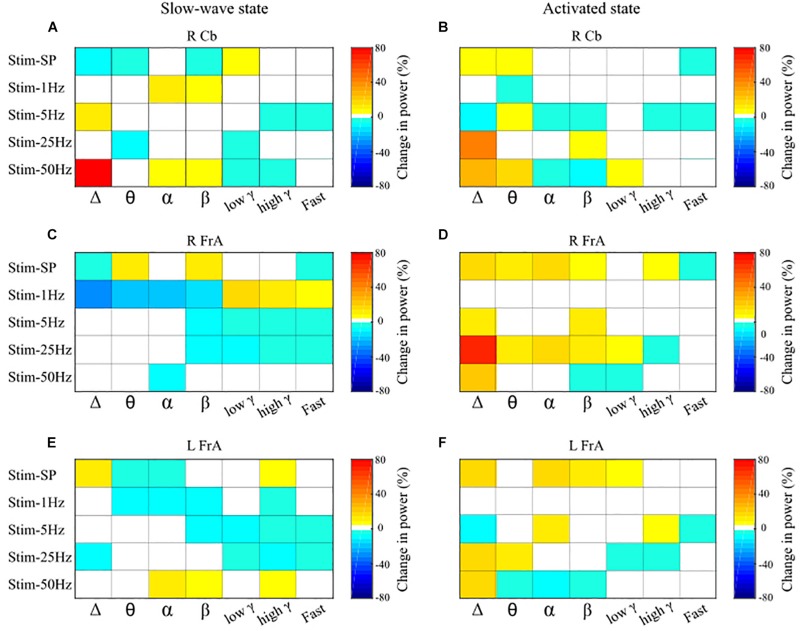
Overall effects of the various stimulation types on power across the different frequency bands, by site. Maximal increases and decreases in mean power within each frequency band induced by stimulation of the cerebellar vermis are summarized here for the different frequencies of stimulation. Panels on the left show changes in mean power relative to the pre-stimulus baseline in the trials when stimulation was delivered during the slow-wave state, and panels on the right show changes in power induced during the activated state. The magnitude of relative changes in power from baseline in percent are represented by the color scales at the right of each panel; white indicates no significant change. Results are shown for the power of LFP activity in the right cerebellum (RCb; **A,B**), right frontal association area (RFrA; **C,D**), and left frontal association area (LFrA; **E,F**). Note the different patterns of changes in power induced by cerebellar stimulation that were dependent upon the presence of either the slow-wave or the activated state. SP, single-pulse; Δ, delta; θ, theta; α, alpha; β, beta; γ, gamma.

Overall, the stimulation patterns affected power to a greater extent than coherence. Stimulation at 1 Hz had larger effects when delivered during the slow-wave state (Stage 1), while single-pulse, 25 and 50 Hz stimulation had stronger effects in the activated state (Stage 2). Figure 4 indicates maximal post-stimulus changes in power in each frequency band induced by each stimulation frequency for all sites; the left panels show power changes during slow-wave activity, and those on the right show changes during the activated state. Relative % change for the different stimulation patterns, in numerical values, are given in the Supplementary  Material.

#### Single-Pulse Stimulation

Single-pulse stimulation (slow-wave: *n* = 10 trials from 4 rats; activated state: *n* = 8 trials from 3 rats) had several effects on power in cerebellar and cortical sites, but no significant effect on cerebello-cortical or cortico-cortical coherence. In general, single-pulse stimulation had a more robust effect on LFP power when delivered in the activated state (Stage 2). Single-pulse stimulation in Stage 2 resulted in increases in power in the Δ and θ bands in the cerebellum, and in a broader range of frequency bands in cortical sites (up to low γ in the LFrA and up to high γ in the RFrA). On the other hand, single-pulse stimulation during Stage 1 activity resulted in mixed effects on power in all sites. Maximal changes in power ranged between 5 and 15% from baseline (see Figure 4, Stim-SP).

Statistical comparisons showed main effects of Stage in Δ [*F*_(1, 39)_ = 11.21, *p* = 0.002], θ [*F*_(1, 39)_ = 6.65, *p* = 0.014], and β [*F*_(1, 39)_ = 5.04, *p* = 0.031], with Stage 2 being affected to a greater extent in all cases. During Stage 1, Δ was reduced in the cerebellum and in the RFrA, but during Stage 2, Δ power was increased in all sites. The main effect of stage in θ and β, was also due to increases in power during Stage 2, especially for the RFrA. Single pulse stimulation therefore induced the greatest increases in cortical power when delivered in the activated state, in a wide range of frequencies (Figures 4D,F). There was also a main effect of site in Δ [*F*_(2, 39)_ = 3.87, *p* = 0.029], θ [*F*_(2, 39)_ = 4.80, *p* = 0.014], and β [*F*_(2,39)_ = 3.75, *p* = 0.032], with the RFrA showing the greatest changes. Overall, there were more increases in power in the RFrA following single-pulse stimulation, and those increases were mainly in the activated state.

#### 1 Hz Stimulation

Stimulation at 1 Hz had a much stronger effect on power during the slow-wave state than during the activated state. There were several changes in power in all sites in Stage 1 but very few in Stage 2 (Figure 4, Stim-1 Hz). We did not find any main effects or interactions in the ANOVA for coherence.

**FIGURE 5:**
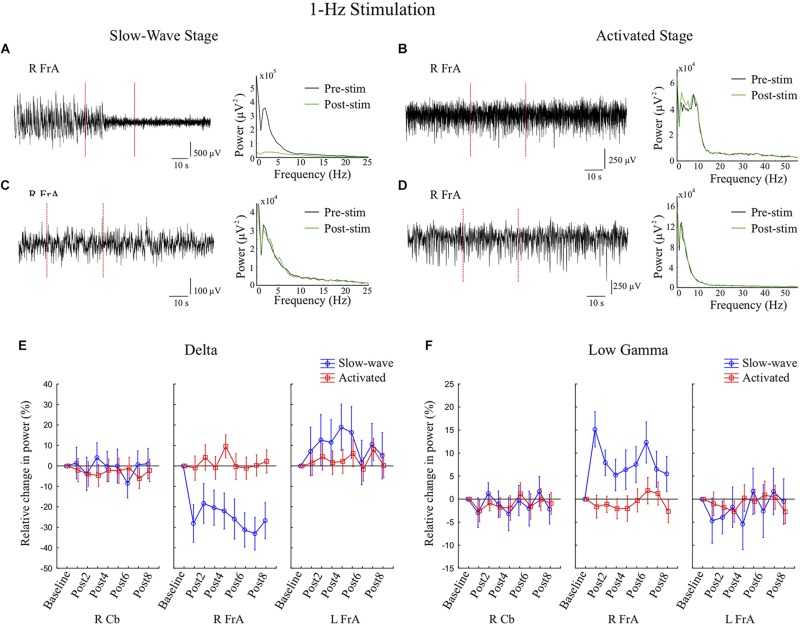
Stimulation at 1 Hz for 30 s during the slow-wave state decreases delta activity and increases low gamma activity in the right frontal association area (RFrA). **(A,C)** Examples are shown in which 1 Hz stimulation (during the period between the vertical red dashed lines) was followed by either greatly reduced slow-wave activity (A, rat 2), or a more moderate reduction in slow-wave activity (**C**, rat 6). Power spectra show corresponding reductions in power in the delta band from pre-stimulation (Pre-stim, black line) to post-stimulation (Post-stim, green line). **(B,D)** During the activated state, 1 Hz stimulation did not significantly affect power in the delta band in the RFrA. Examples of LFP traces in the RFrA and corresponding power spectra are shown for two animals (**B**, rat 2; **D**, rat 4) in which there were minimal changes post-stimulation. **(E,F)** The mean percent changes in power relative to baseline are shown for the group of animals (slow-wave: *n* = 7 trials from 4 rats; activated state: *n* = 14 trials from 6 rats) for the delta band **(E)** and for the low gamma band **(F)** for all three recording sites. Results are shown for the eight 6 s time windows following stimulation (Post1–Post8). The reduction in power in the delta band, and the increase in power in the low gamma band occurred only during the slow-wave state (blue lines) in the RFrA.

1 Hz stimulation during Stage 1 increased power in the RCb in the α and β bands, but decreased RCb θ power in Stage 2. In Stage 1, there were decreases in θ, α, β and high γ in the LFrA. In the RFrA, power in slow frequencies (Δ, θ, α, and β) decreased, while power in faster frequencies (low γ, high γ, and Fast) increased (Figure 4C). These changes ranged between 33% decreases (in Δ) and 15% increases (low γ). Figure 5 shows example LFP traces and power spectra of 1 Hz stimulation trials in the slow-wave state (Figures 5A,C), examples in the activated state (Figures 5B,D), and the mean percent changes in power relative to baseline in the delta (Figure 5E) and low gamma bands (Figure 5F) for the group of rats (slow-wave: *n* = 7 trials from 4 rats; activated state: *n* = 14 trials from 6 rats). These state-dependent effects on power were supported by statistically significant Stage by site interactions in the Δ band, with the RFrA showing a reduction during Stage 1 [*F*_(2, 45)_ = 5.31, *p* = 0.009], and in the θ band, with reductions in the R and LFrA during Stage 1 [*F*_(2, 45)_ = 3.52, *p* = 0.038]. Overall, the RFrA showed the greatest change between stages. This implies that 1 Hz stimulation during the slow-wave state can shift LFP activity to higher frequencies, decreasing 0–30 Hz activity while increasing activity in the 30–200 Hz range.

#### 5 Hz Stimulation

5 Hz stimulation (slow-wave: *n* = 8 trials from 3 rats; activated state: *n* = 8 trials from 4 rats) led to marked changes in power during both Stage 1 and Stage 2, but there were no main effects of stimulation or interactions for the coherence measures. In Stage 1, stimulation at 5 Hz led mainly to decreases in power, especially in high frequency bands (β, low γ, high γ, and Fast for cortical sites; high γ, and Fast for the RCb). During Stage 2, there were both increases and decreases in power at different recordings sites distributed across all frequency bands (Figure 4, Stim-5 Hz**).** There was a main effect of time for the low γ band [*F*_(8, 288)_ = 2.47, *p* = 0.013], due to a more pronounced drop in power at Post4, indicating a strong decrease in 30–55 Hz oscillatory power about 25 s post-stimulus. There also was a Stage by time interaction for β [*F*_(8, 288)_ = 2.12, *p* = 0.034] with greater power during Stage 2 than during Stage 1 at all delays except Post3; Stage 2 had an increase in β power while Stage 1 had a decrease. Overall, stimulation at 5 Hz decreased high frequency (15–200 Hz) power in cortical sites during slow-wave activity.

#### 25 Hz Stimulation

**FIGURE 6:**
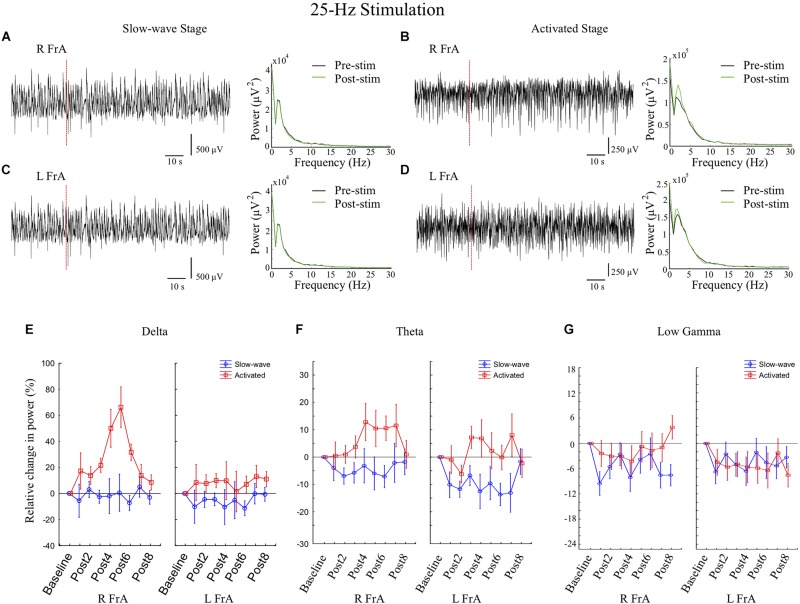
In cortical sites, stimulation at 25 Hz for 2 s increases power in low frequency bands in the activated state and decreases low gamma activity in the slow-wave state. **(A,C)** During the slow-wave state, representative examples of local field potential (LFP) traces and power spectra in the right frontal association area (RFrA) **(A)** and LFrA **(C)** showing no marked changes in response to 25 Hz stimulation (vertical red dashed line; rat 5) as clear peaks in the power spectrum at high frequencies were rarely visible. **(B,D)** During the activated state, LFP traces and power spectra in the RFrA **(B)** and LFrA **(D)** showed increased power in the delta band following stimulation (Post-stim, green line) compared to pre-stimulation (black line; rat 4). **(E–G)** The mean percent changes in power in cortical sites relative to baseline are shown for the group of animals (slow-wave: *n* = 7 trials from 4 rats; activated state: *n* = 7 trials from 5 rats) for the delta band **(E)**, theta band **(F)**, and low gamma band **(G)**. Results are shown for the eight 6 s time windows following stimulation (Post1–Post8). Increases in power in the delta and theta bands occurred in the activated state (red line), while reductions in power in the low gamma band were more reliable in the slow-wave state (blue line).

Stimulation at 25 Hz induced greater effects on power and coherence during the activated state. During Stage 2, there were large increases in the Δ band in all sites (66% in the RFrA, 13% in the LFrA, and 41% in the RCb). Power also increased in θ in the LFrA and in θ, α, β, and low γ in the RFrA. In Stage 1, stimulation at 25 Hz decreased cortical power in high frequency bands (LFrA: 30–200 Hz; RFrA: 15–200 Hz). 25 Hz stimulation therefore had strong, state-dependent effects on LFP activity in cortical sites, with an increase of lower frequency activity in the activated state and a decreased activity in faster bands in the slow-wave state (Figures 4C–F, Stim-25 Hz). These state-dependent effects of 25 Hz stimulation are illustrated in Figure 6 where example LFP traces and power spectra in both stages (Figures 6A–D), and the mean percent changes in power relative to baseline in the delta (Figure 6E), theta (Figure 6F), and low gamma bands (Figure 6G), for the group of rats (slow-wave: *n* = 7 trials from 4 rats; activated state: *n* = 7 trials from 5 rats), are shown.

State-dependent effects of 25 Hz stimulation were reflected by main effects of Stage in Δ [*F*_(1, 30)_ = 13.81, *p* = 0.001], θ **[***F*_(1, 30)_ = 10.78, *p* = 0.003], β [*F*_(1, 30)_ = 5.96, *p* = 0.021], and Fast [*F*_(1, 30)_ = 5.56, *p* = 0.025], with Stage 2 being higher in all cases. A Stage by time interaction in Δ [*F*_(8, 240)_ = 2.21, *p* = 0.028], with greater increases in power during Stage 2 at multiple time points (Post3–6), also indicates the sensitivity of the activated state to 25 Hz stimulation. There was a main effect of time in β [*F*_(8, 240)_ = 2.44, *p* = 0.015], low γ [*F*_(8, 240)_ = 1.98, *p* = 0.049], high γ [*F*_(8, 240)_ = 3.68, *p* = 0.001], and Fast [*F*_(8, 240)_ = 4.33, *p* = 0.00007] frequency bands with mostly decreases post-stimulus, meaning that there was a general decrease in high frequency power after stimulation. A time by site interaction in Δ [*F*_(16, 240)_ = 2.33, *p* = 0.003] was also present, with the cerebellar site showing an early increase, and the RFrA showing a later increase.

Analysis of changes in coherence following 25 Hz stimulation showed a main effect of Stage in β [*F*_(1, 24)_ = 6.61, *p* = 0.017], with greater increases in coherence during Stage 2. We also found a Stage by Comparison effect in β [*F*_(2, 24)_ = 4.84, *p* = 0.017] in which the contra Cb-FrA comparison showed the greatest difference between Stages. 25 Hz stimulation therefore induced larger increases in coherence in the activated state, especially in the contra Cb-FrA comparison where coherence was increased during Stage 2 and decreased during Stage 1. This shows that 25 Hz stimulation can entrain and synchronize activity in the β band in cerebello-cortical networks when delivered in the activated state.

#### 50 Hz Stimulation

Overall, stimulation at 50 Hz (slow-wave: *n* = 9 trials from 4 rats; activated state: *n* = 9 trials from 5 rats) had a greater effect on power during the activated state. Once again, more effects were noted for power than for coherence. There were increases in Δ power in all sites in Stage 2 and a large increase in the RCb (80%) in Stage 1. Power also increased in α, β, and high γ in the LFrA in Stage 1, while it decreased in the 3–30 Hz range in Stage 2 (Figure 4, Stim-50 Hz).

There was a main effect of Stage in Δ [*F*_(1, 36)_ = 6.47, *p* = 0.015] with increases in power being greater in Stage 2, suggesting that the activated state was the most responsive within the 0–3 Hz range. A significant Stage by time interaction in Δ [*F*_(8, 288)_ = 2.11, *p* = 0.035] showed that power increased at Post1 in both stages, but then decreased slightly below baseline in Stage 1 while remaining elevated in Stage 2. There was also a time by site interaction in Δ [*F*_(16, 288)_ = 2.06, *p* = 0.010], indicating the cerebellar site was affected earlier and more strongly by the stimulation. We also saw a Stage by site interaction for low γ [*F*_(2, 36)_ = 4.18, *p* = 0.023], in which the power in the cerebellum was also affected more strongly than cortical sites. This implies that the effects 50 Hz stimulation differed the most as a function of stage in the cerebellar site.

Analysis of coherence showed that there was a main effect of time in high γ [*F*_(8, 248)_ = 2.49, *p* = 0.013], with an early increase, followed by a slight decrease in coherence. When exploring specific Stage by site by frequency interactions for high γ, it was found that ipsi Cb-FrA and FrA-FrA coherence increased and contra Cb-FrA coherence decreased following 50 Hz stimulation.

## Discussion

The cerebellum is thought to play an important role in cognitive function through its interactions with the prefrontal cortex (Hoppenbrouwers et al., 2008; Strick et al., 2009; Bostan et al., 2013). Both slow and fast oscillatory rhythms are thought to coordinate interactions between the cerebellum and cortical sites (O’Connor et al., 2002; Courtemanche and Lamarre, 2005; Ros et al., 2009; Courtemanche et al., 2013; Popa et al., 2013; Chen et al., 2016), and rhythmic cerebellar stimulation has been used as a therapeutic intervention in some disorders (Schutter et al., 2003; Schutter and van Honk, 2006, 2009; Demirtas-Tatlidede et al., 2010). The present study has examined the effects of cerebellar vermal stimulation at various rhythms on the entrainment of cerebellar and cortical LFPs under urethane anesthesia. Our results show that there are frequency-specific effects of cerebellar stimulation on both cerebellar and cerebral cortical LFP spectral properties, and that cerebellar stimulation at high frequencies (25 and 50 Hz) can also promote coherence in this cerebello-cortical network. Our findings also indicate that the effects of vermal stimulation are highly dependent upon the initial state of the networks, and that markedly different patterns of results were obtained, particularly for cortical sites, when stimulation was applied during the slow-wave versus the activated state.

Cerebellar vermal stimulation during either the slow-wave state or activated state produced different effects on cerebellar hemispheric LFPs. Single-pulse and 50 Hz stimulation led to opposite changes in LFP power when delivered during the slow-wave state as opposed to the activated state (Figures 4A,B). For this site, stimulation at a low rate would produce variable effects on the slower frequency bands; at higher rates, the effect was mostly to decrease the power at low gamma frequency and higher. This effect was clear for the 5, 25, and 50 Hz stimulations, and was most potent during slow-wave activity. There was also an effect of the 25 and 50 Hz stimulation in increasing power in the delta band.

Stimulation of the vermis induced markedly different effects on the prefrontal cortex LFPs depending on the initial state. In the slow-wave state, 5 and 25 Hz stimulation induced a strong decrease in power in the beta to Fast frequency bands in both the right and left FrA. Stimulation at 1 Hz during the slow-wave state also had a strong effect: delta-to-beta activity decreased, while the low gamma-to-fast activity increased in the right FrA. In the activated state, however, stimulation using single pulses, and at 5, 25, and 50 Hz resulted in an overall increase in power across the delta-to-beta bands.

### State-Dependent Effects of Stimulation

One of the main findings in our study is that the effects of stimulation were influenced by the stage of urethane anesthesia (Clement et al., 2008). This highlights the importance of the initial oscillatory state in determining the susceptibility of target structures for changes in LFP oscillations and entrainment within different frequency bands. Previous research investigating the effects of vermal stimulation on frontal oscillations in humans, cats, and rodents showed that low frequency stimulation mainly affects slow activity, while stimulating at higher frequencies increased activity in faster bands (Steriade, 1995; Schutter et al., 2003; Schutter and van Honk, 2006; Parker et al., 2017). Experiments reported here used various stimulation frequencies, and demonstrated a range of effects that were dependent on baseline oscillatory state.

#### Pathways Mediating the Effects of Stimulation

Reciprocal anatomical connections have been well established between the cerebellum and prefrontal cortex, via cerebello-thalamo-cortical and cortico-ponto-cerebellar pathways (Kelly and Strick, 2003; Strick et al., 2009; Watson et al., 2009, 2014; Buckner et al., 2011; Farzan et al., 2016), but how rhythmic cerebellar output modulates cortical activity is still an open question. The stimulation in the cerebellar vermis, in reaching the prefrontal cortex, likely coursed through the fastigial nucleus and then to the thalamus (Bostan et al., 2013; Lisberger and Thach, 2013). Stimulation of Purkinje cells, in the outermost layer of the cerebellum, leads to changes in the cerebellar output, which in turn modulates the output of deep cerebellar nuclei (DCN) (Oulad Ben Taib and Manto, 2013, 2016; Das et al., 2017). Because inputs from Purkinje cells to the DCN are inhibitory, increased activation of Purkinje cells with high frequency stimulation (Maeda et al., 2000b; Hallett, 2007) inhibits the tonic activity of the DCN. This would in turn decrease excitation in the thalamus. However, it is also quite possible that DCN neurons could also show rebound excitation (Buzsaki, 2006). Subsequent activation of extra-cerebellar areas via the thalamus may thus occur through rebound excitation within the DCN (Buzsaki, 2006; Hoebeek et al., 2010). This phenomenon has been reported mainly in thalamic, cortical, and DCN neurons (Grenier et al., 1998; Buzsaki, 2006; Hoebeek et al., 2010; Boehme et al., 2011). After the initial inhibition induced by stimulation, the T-channel is activated causing Ca^2+^ influx, which leads to a slow rebound spike. Thus, the initial hyperpolarization of the fastigial nuclei, induced by electrical stimulation of the vermis, would lead to a burst of rebound spikes in the DCN up to 100 ms after the hyperpolarization ceases. If these spikes occur in synchrony and interact with the necessary opposing currents (mixed cation current, I_*h*_), oscillations could be generated and would then propagate to thalamocortical pathways (McCormick and Pape, 1990; Buzsaki, 2006). The emergence of different oscillatory patterns thus depends not only on the strength and frequency of the applied stimuli, but also on factors regulating the intrinsic excitability and rhythmicity of neurons. Indeed, in this mode, when the effects of stimulation on oscillations rely on rebound excitation mechanisms (high frequency stimulation), the initial state of the neuron strongly impacts the effects of inputs (Buzsaki, 2006). This is in line with the state-dependent effects of stimulation that we have found here, with stimulation at high frequencies (25 and 50 Hz) leading to greater changes when initially in the activated state, and stimulation at 1 Hz inducing more effects in the slow-wave state.

Low frequency stimulation on the other hand may cause a decrease in activity of Purkinje cells (Chen et al., 1997), which would reduce inhibitory input to the fastigial nucleus, and result in greater excitatory drive to the thalamus. In a study investigating responses to different types of stimulation, single-pulse stimulation of the cerebellar cortex (paravermal lobules VI/VII) increased the chance of spiking for a short period post-stimulation (after a latency of ∼8 ms), but did not alter the firing frequency of DCN neurons (Hoebeek et al., 2010). Although the mechanisms are not fully understood, this effect on spike timing occurred in the absence of rebound excitation.

In this study, vermal stimulation could entrain cerebello-cortical networks. However, given the duration of the changes observed, stimulation was unlikely to have induced long-term potentiation (LTP). For instance, high frequency stimulation (100 Hz in bursts of 15 pulses, for a total of 1500 pulses) applied to the parallel fibers, in the most superficial layer of the cerebellar cortex, has been shown to induce LTP at synapses between parallel fibers and Purkinje cells (Jörntell and Ekerot, 2002). Therefore, although we did not assess induction of LTP in this study, the number of pulses delivered in our study was likely too low to lead to lasting plastic changes.

#### Cortical Effects

Our results show that the effects of stimulation are state-dependent. Indeed, LFP activity fluctuates in urethane-anesthetized rats in cyclic alternations that are similar to sleep stages (Clement et al., 2008). When applied in the slow-wave state, the faster (5, 25, and 50 Hz) stimulations produced a noticeable decrease in cortical power for the faster frequency bands. In the activated state, the same stimulations produced an overall increase in power across the slower bands. The bands most affected were thus markedly different between the two states. The influence of the initial brain state on the effects of stimulation has been investigated in humans, in studies using TMS or direct cortical stimulation, as well as in rats (Jackson et al., 2008; Alagapan et al., 2016; Connolly et al., 2016; Silvanto et al., 2017).

The effects of the stimulation at 1 and 25 Hz provide good examples of this modulation by state. During the slow-wave state, 1 Hz stimulation would increase power in the 30–200 Hz range, while decreasing power in the 0–30 Hz band, however, 1 Hz stimulation did not show any effects in cortical sites during the activated state. The effects produced by 1 Hz stimulation can be interpreted partially by the mechanism of generation of slow-wave activity in thalamocortical networks, modulated by thalamic neuronal activity. Delta activity in the brain, sleeping and anesthetized, stems from an interaction between thalamic and cortical oscillators (Steriade, 2003). Optogenetic stimulation of thalamocortical neurons at 1 Hz triggers their firing of bursts of action potentials, and is also an optimal frequency for inducing cortical slow waves; stimulations at 1.5 Hz or higher on the other hand failed to entrain EEG activity (David et al., 2013). It is possible that 1 Hz stimulation of the cerebellar cortex in our recordings disrupted thalamic mechanisms that mediate delta activity, in a manner specific to the slow-wave state. This could decrease activity in the delta range while increasing activity in faster frequency bands. Optogenetic stimulation of cerebellar projections at 2 Hz was also shown to re-establish normal levels of delta activity in an awake rat model of schizophrenia, which shows lower delta activity in the medial frontal cortex similar to observations in schizophrenic patients (Parker et al., 2017). The threshold stimulation frequency for the cerebellar cortex to entrain delta activity would likely then be affected by the initial state in the cerebral cortex. As our results show, the initial state strongly affects the optimal stimulation pattern for entrainment at various frequencies.

Conversely, in the slow-wave state, 25 Hz stimulation produced a decrease of power in a wide range of higher frequencies (15–200 Hz), while inducing a strong increase in the 0–55 Hz band in cortical sites during the activated state. This can be compared to the work of Steriade (1995), who used 300 Hz stimulation of the fastigial nucleus in ketamine/xylazine anesthetized cats, and showed an attenuation of slow rhythms, and an enhancement of 20–40 Hz oscillatory activity in the frontal cortex. We did not find this strong effect of high-frequency stimulation in decreasing slow-wave activity in our recordings, but we did find an increase in beta/gamma power following 25 Hz stimulation in the activated state. In addition, using stimulation at 100–200 Hz of the brachium conjunctivum (i.e., afferents to the thalamus from the cerebellar nuclei), the same team (Timofeev and Steriade, 1997) found an activation of the cat EEG at 30–100 Hz during ketamine/xylazine anesthesia. Again, we found a similar increase in power in these bands only during 25 Hz stimulation in the activated state. Differences in these results could be due in part to the type of anesthetic used, or to the different axon conduction speed and synaptic delays characteristic of different species (Buzsáki et al., 2013). Differences could also be due to the much higher stimulation frequencies used in those studies (Steriade, 1995; Timofeev and Steriade, 1997). In the rat, in order to increase motor cortical excitability, stimulation of the lateral cerebellar nucleus at different frequencies showed a greater facilitation at 30 Hz, similar to our effects at 25 Hz in the activated state (Baker et al., 2010). Similarly, stimulation of the same nucleus at 30–50 Hz increased contralateral cortical excitability, measured as motor evoked potentials, in a rat model of stroke (Park et al., 2015). It would be interesting to monitor the oscillatory state differences prior to stimulation in these awake animals, as it could have played a role in the optimal responsivity to stimulation. Overall, it does appear that prefrontal cortex networks can generate and resonate with beta and gamma rhythms and that activity in these bands can be modulated by cerebellar output (Sherfey et al., 2018).

#### Cerebellar Effects and Minimal Effects on Cerebello-Cortical Coherence

We assessed here whether different frequencies of patterned stimulation can entrain cerebellar frequency-specific patterns that have been previously described (Courtemanche et al., 2013). In the slow-wave state, stimulation at 1 and 50 Hz produced more effects on cerebellar oscillatory activity. These stimulation frequencies caused an augmentation of 8–30 Hz power, which corresponds to the range of oscillatory activity in the granule cell layer (GCL) of the cerebellum, and this effect could have been mediated directly, or through pathways projecting to the GCL such as the parallel fibers. In addition, stimulation at 50 Hz also caused a strong increase in delta power, which could be due in part by the brief nature of this stimulation train, which may have activated multiple cerebellar units in-phase, likely through parallel fibers, especially if the stimulation was timed with the ascending phase or peak of a slow rhythm. In the activated state, effects within the 8–30 Hz range were mostly absent, but stimulations at 25 and 50 Hz had strong effects on the 0–8 Hz activity in the cerebellum. This could be again due to a phasic increase in excitation timed with a slow cerebellar rhythm. The 5 Hz stimulation also increased power in the theta band, potentially affecting a theta-related oscillatory pattern already present in the cerebellum (Courtemanche et al., 2013).

Our results showed significant effects of stimulation mainly for measures of power across frequency bands, and less so for coherence measures. Synchronized activity between the cerebellum and cortex has been observed in a variety of contexts. Coherent activity in the alpha and beta frequency ranges occurs in the cerebellum and sensorimotor cortex during actions requiring somatosensory monitoring (O’Connor et al., 2002; Courtemanche and Lamarre, 2005). Functionally as well, synchronization of LFPs between the medial prefrontal cortex and the cerebellum at 5–12 Hz has been linked with adaptive performance in eyeblink conditioning during the early stages of learning (Chen et al., 2016). Multiple brain regions, including the amygdala, hippocampus, medial prefrontal cortex, and cerebellum must coordinate to acquire a variety of learned responses, such as the conditioned eyelid response in the eyeblink conditioning paradigm (Lee and Kim, 2004). Coherent activity between the cerebellum and prefrontal cortex across a variety of bands may thus contribute to acquiring appropriate behaviors through associative learning and during performance. There are other clear indications that the cerebellum is important in cortical synchronization (Courtemanche et al., 2013). The functional role of the cerebellum in gamma-band coherence between areas of the cerebral cortex has been demonstrated in rats; inactivating the cerebellum with a muscimol injection disrupted cortical coherence in gamma between the sensory and motor cortices, potentially interrupting transmission of sensorimotor information between these areas (Popa et al., 2013). Finally, a recent study also showed Purkinje cell simple spike timing is related to coherent cerebral cortical oscillatory activity (McAfee et al., 2019).

Why did we not find clear effects on coherence? An obvious first consideration is the anesthetic state. The anesthetic state carries with it clearly different patterns of large-scale oscillations and coherence than the awake state (Steriade, 2003), but urethane anesthesia has been shown to be permissive to network oscillations (Maggi and Meli, 1986; Clement et al., 2008; Frederick et al., 2014; Robinson et al., 2017). Coherent activity in our preparation was abundant (see Figure 3), and the coherent slow-wave state represented a majority of the total duration of our recordings (e.g., see Figure 2). Ros et al. (2009) have shown that the cerebellum can generate slow oscillations that are synchronized with those of the neocortex, and that neocortical oscillations drive cerebellar rhythms. The strong slow-wave state throughout the recordings may have hindered our capacity to detect coherence effects. The state of the network in this study was likely similar to a resting-state condition, first described in idling states and during early stages of sleep in fMRI studies, but also in the anesthetized state in humans, monkeys, and rodents (Lu et al., 2007; Raichle, 2015). It is thus quite possible that during anesthesia, as in sleep, large-scale coherent slow-wave mechanisms protect the cortical circuits from outside disturbance via the thalamocortical circuit isolation properties (Steriade, 2003), and that this may reduce effects of stimulation on coherence. It is possible that over our particular anesthesia modes, coherent activity between sites acts as a filtering mechanism to suppress external inputs, as happens during functional inhibition to optimize performance (Courtemanche et al., 2003; Jensen and Mazaheri, 2010). Another consideration is that the placement of our frontal lobe recording electrodes and/or our cerebellar stimulation electrodes was perhaps not optimal to evaluate between-site synchrony. The methodological approach of evaluating the location of cerebral cortical best response to stimulation through evoked potentials could help determine an optimal electrode alignment (Watson et al., 2009, 2014) and might further help in finding coherent sites.

### Effects of Cerebellar Stimulation on Frontal Cortical Networks

Cerebro-cerebellar loops involving prefrontal cortical areas have received increased attention over the last few decades. The initial explorations concerned cerebro-cerebellar relationships in sensorimotor circuits (Allen and Tsukahara, 1974; Sasaki, 1979; Bloedel and Courville, 1981; Morissette and Bower, 1996; Courtemanche and Lamarre, 2005). In parallel, the identification of cognitive roles for the cerebellum was being progressively characterized through neuropsychological testing and studies in patients (Leiner et al., 1991; Akshoomoff and Courchesne, 1992; Courchesne et al., 1994; Akshoomoff et al., 1997; Mangels et al., 1998). Advances in brain imaging also indicated a cerebellar role in cognition (Roland, 1993; Allen et al., 1997, 2005; Schmahmann, 1997; Allen and Courchesne, 2003; Buckner, 2013; Schmahmann, 2019). Anatomical reports showed precise functional connections between the cerebellum and prefrontal cortex in the primate (Schmahmann and Pandya, 1997a, b; Kelly and Strick, 2003) which could mediate cerebro-cerebellar loops involved in cognitive operations. The cerebello-cerebral connectivity displays multiple parallel loops mediating processes related to sensation, movement, and thought (Middleton and Strick, 2000; Strick et al., 2009; Bostan et al., 2013; Bostan and Strick, 2018). Even though the nature of the prefrontal cortex in rodents is the object of some debate, multiple cognitive and executive functions are performed by prefrontal cortex or similar regions in rats (Kolb, 1984; Uylings et al., 2003; Dalley et al., 2004; Kesner and Churchwell, 2011; Leonard, 2016). Cerebello-prefrontal cortex connectivity has also been confirmed using physiological and anatomical measures (Suzuki et al., 2012), and has been explored electrophysiologically, by evaluating cerebellar evoked potential and cellular responses obtained through medial prefrontal cortical stimulation, as well as through measures of prefrontal cortex neurophysiological activity following fastigial nucleus stimulation (Watson et al., 2009; Watson et al., 2014). In addition, related to the results presented here, Watson et al. (2014) found a cerebello-cerebral directed coherence pattern in the theta range that was prominent during active locomotion, showing that these connections could support cerebello-cerebral communication. Our current study has contributed to understanding how different frequencies of cerebellar oscillations modulate oscillations and coherent activity within cerebello-cortical networks (Courtemanche et al., 2013).

The effects of cerebellar stimulation on the activity of cerebello-prefrontal loops remains largely unexplored in the rat, in which the underlying neuronal pathways and mechanisms can be assessed. Our findings show that even under anesthesia, cerebello-cortical network interactions can be modulated through cerebellar stimulation. A multi-site multi-electrode approach could enhance the fine-grained mapping through evoked responses and/or unit activity to study the spatio-temporal properties of cerebello-cortical connectivity. Such an approach would also allow changes in evoked synaptic responses to be monitored in association with ongoing EEG rhythms and state (Ozen et al., 2010; Marquez-Ruiz et al., 2014), to provide some insight into the strength of synaptic pathways during oscillations (Timofeev et al., 1996; Rosanova and Timofeev, 2005). Future studies could also investigate the effects of cerebellar stimulation in awake animals, during behavior or rest. These would require the study of oscillatory and synchronous networks at smaller timescales, with analytical methods able to follow fast changes in network configuration, such as phase synchrony analysis (Lachaux et al., 1999). The initial oscillatory or activation state can be expected to strongly impact the effects of stimulation. In the activated state, the cerebellar stimulation with single-pulses, as well as with repeated pulses at 25 Hz, was optimal in generating increased delta-gamma band activity, which could correspond to an analog of cortical cross-frequency entrainment (Helfrich et al., 2014, 2016). It would also be interesting to test the physiological effects of cross-frequency coupled nested rhythms in the awake-behaving animal. As the cerebellum contributes to higher-order functions, understanding how cerebello-cerebral loops operate and respond to stimulation is essential in uncovering the underlying physiology, but also in developing new methods to address numerous disorders.

## Data Availability Statement

The datasets generated for this study are available on request to the corresponding author.

## Ethics Statement

The animal study was reviewed and approved by Concordia University Animal Research Ethics Committee.

## Author Contributions

ST, CC, and RC designed and prepared the experiments, acquired the data, and wrote the manuscript. ST and RC analyzed the data.

## Conflict of Interest

The authors declare that the research was conducted in the absence of any commercial or financial relationships that could be construed as a potential conflict of interest.
