# Supplementary material for: State-Dependent Entrainment of Prefrontal Cortex Local Field Potential Activity Following Patterned Stimulation of the Cerebellar Vermis
Source: Front Syst Neurosci. 2019 Oct 29;13:60. doi: 10.3389/fnsys.2019.00060 (PMC6828963; doi:10.3389/fnsys.2019.00060)
Supplement: Supplementary file 1 [file Data_Sheet_1.PDF]

Supplementary Material

Tables of Changes in Power (% change from baseline) following stimulation

Legend:

Stage 1 / Stage 2

Δ= delta; θ= theta; α= alpha; β= beta; γ= gamma

Single-pulse stimulation

Table 1. Changes in power (% change from baseline) following Single-pulse stimulation when delivered in the slow-wave state.

Site	Δ	θ	α	β	low γ	high γ	Fast
R Cb	-10.8	-6.1	0	-4	+3	0	0
R FrA	-5.9	+8.4	0	+10.7	0	0	-3.9
L FrA	+12.9	-6.9	-6.9	0	0	+4.4	0

Table 2. Changes in power (% change from baseline) following Single-pulse stimulation when delivered in the activated state.

Site	Δ	θ	α	β	low γ	high γ	Fast
R Cb	+5	+6.3	0	0	0	0	-3.8
R FrA	+15.1	+9.7	+13.1	+7.7	0	+5.2	-3.6
L FrA	+14.3	0	+13	+8.8	+4.8	0	0

1-Hz Stimulation

Table 3. Changes in power (% change from baseline) following 1-Hz stimulation when delivered in the slow-wave state.

Site	Δ	θ	α	β	low γ	high γ	Fast
R Cb	0	0	+8.2	+4.8	0	0	0
R FrA	-33.1	-20.8	-19.1	-16.3	+15.2	+10.2	+7
L FrA	0	-12.8	-12.2	-9.4	0	-6.6	0

Table 4. Changes in power (% change from baseline) following 1-Hz stimulation when delivered in the activated state.

Ch	Δ	θ	α	β	low γ	high γ	Fast
R Cb	0	-6.8	0	0	0	0	0
R FrA	0	0	0	0	0	0	0
L FrA	0	0	0	0	0	0	0

5-Hz Stimulation

Table 5. Changes in power (% change from baseline) following 5-Hz stimulation when delivered in the slow-wave state.

Site	Δ	θ	α	β	low γ	high γ	Fast
R Cb	+9.2	0	0	0	0	-4.8	-3.6
R FrA	0	0	0	-9.8	-5.7	-6.6	-5.3
L FrA	0	0	0	-10.2	-9.3	-3.9	-4.8

Table 6. Changes in power (% change from baseline) following 5-Hz stimulation when delivered in the activated state.

Site	Δ	θ	α	β	low γ	high γ	Fast
R Cb	-9.2	+6.5	-5.3	-5.4	0	-4.8	-4.5
R FrA	+9.2	0	0	+8.8	0	0	0
L FrA	-11.8	0	+9.5	0	0	+5.1	-3.5

25-Hz Stimulation

Table 7. Changes in power (% change from baseline) following 25-Hz stimulation when delivered in the slow-wave state.

Site	Δ	θ	α	β	low γ	high γ	Fast
R Cb	0	-9.8	0	0	-5.4	0	0
R FrA	0	0	0	-9.2	-9.6	-6.8	-4.6
L FrA	-11.3	0	0	0	-6.8	-8.2	-5.3

Table 8. Changes in power (% change from baseline) following 25-Hz stimulation when delivered in the activated state.

Site	Δ	θ	α	β	low γ	high γ	Fast
R Cb	+40.6	0	0	+6.7	0	0	0
R FrA	+66.2	+12.8	+17.1	+10.9	+3.8	-7.4	0
L FrA	+13	+8	0	0	-7.6	-6.4	0

50-Hz Stimulation

Table 9. Changes in power (% change from baseline) following 50-Hz stimulation when delivered in the slow-wave state.

Site	Δ	θ	α	β	low γ	high γ	Fast
R Cb	+80	0	+5.5	+5.5	-5	-4.9	0
R FrA	0	0	-9.6	0	0	0	0
L FrA	0	0	+12.4	+6.7	0	+7.3	0

Table 10. Changes in power (% change from baseline) following 50-Hz stimulation when delivered in the activated state.

Site	Δ	θ	α	β	low γ	high γ	Fast
R Cb	+27.9	+14.4	-7.5	-8.7	+3.3	0	0
R FrA	+22	0	0	-6	-5.9	0	0
L FrA	+17.2	-6.9	-12.1	-7.2	0	0	0
